# Verbal learning impairment in adolescents with methamphetamine use disorder: a cross-sectional study

**DOI:** 10.1186/s12888-021-03169-3

**Published:** 2021-03-25

**Authors:** Lukas Andreas Basedow, Sören Kuitunen-Paul, Melina Felicitas Wiedmann, Stefan Ehrlich, Veit Roessner, Yulia Golub

**Affiliations:** 1 TU Dresden, Faculty of Medicine, Department of Child and Adolescent Psychiatry, Dresden, Germany; 2 TU Dresden, Faculty of Medicine, Division of Psychological and Social Medicine and Developmental Neurosciences, Dresden, Germany

**Keywords:** Crystal meth, Neuropsychology, Stimulants, Learning, Cognitive control, Attention

## Abstract

**Background:**

Methamphetamine (MA) use has been shown to be associated with deficits in impulsivity, verbal learning, and working memory. Additionally, methamphetamine use disorder (MUD) is related to various brain changes, especially in adolescent users who might be more vulnerable to detrimental effects on brain development. However, little is known about the relationship between adolescent MA use and cognitive impairment. This cross-sectional study aims to explore how the presence of a MUD in adolescents is related to impairments of verbal memory, inhibition, and alertness.

**Methods:**

*N* = 18 psychiatric outpatients with MUD were matched in terms of depressivity, age, and gender to *n* = 18 adolescents with other substance use disorders (SUDs), as well as *n* = 18 controls without SUDs. We compared these three groups on the Verbal Learning and Memory Task (VLMT), and the alertness and go/noGo subtests of the Test of Attentional Performance (TAP). Additionally, Spearman’s rank order correlation coefficients were calculated to investigate whether cognitive functioning was directly associated with frequency of past year MA use.

**Results:**

The three groups differed significantly in their verbal learning performance (*H* (2) = 11.7, *p* = .003, η_*p*_^2^ = .19), but not in short-term memory, inhibition, cued recall, or alertness. Post hoc tests revealed significant differences in verbal learning between the MA using group and the control group without a SUD (*U* = 56.5, *p* = .001, η_*p*_^2^ = .31). Frequency of past year MA use correlated negatively with short-term memory (*ρ* = −.25, *p* < .01) and verbal learning (*ρ* = −.41, *p* < .01). No other cognitive variables correlated significantly with MA use frequency. Significant *p*-values were considered significant after Bonferroni correction.

**Conclusions:**

Adolescent MUD outpatients with regular MA use show specific impairment in verbal learning performance, but not in other basal cognitive functions when compared to adolescents without a MUD. Verbal learning and short-term memory performance is negatively associated with the frequency of MA use. Future research should apply longitudinal designs to investigate long-term effects of methamphetamine and reversibility of these effects on cognitive functioning.

**Supplementary Information:**

The online version contains supplementary material available at 10.1186/s12888-021-03169-3.

## Background

Methamphetamine (MA) is a potent psychoactive substance which produces acute stimulating effects and can be consumed via oral ingestion, insufflation, smoking, or intravenous and intramuscular injection [1]. While some individuals use MA in a recreational manner, a large proportion of chronic users develops a stimulant use disorder – methamphetamine type (MUD). For example, in 2012, 1.2 million adults in the U.S.A used MA, of which 44% (535.000) fulfilled the criteria for a MUD [2]. The chronic use of MA, as seen in patients with MUD, is associated with numerous psychological side effects, such as insomnia, agitation, paranoia, acute psychosis, anxiety, and depressive states [3–5]. Furthermore, regular adult MA use was demonstrated to induce mild cognitive impairment [6] and the presence of a MUD seems to be associated with impaired impulsivity, social cognition, verbal learning, and working memory [7].

While a large number of MA users are adults, adolescent use is widespread as well. In Europe, the prevalence of MA use in high school students (15–16 years) is highest in Poland and Cyprus, with 2.4 and 2.5% respectively, while in Germany prevalence of MA use is estimated to be around 0.7% [8]. Like adult users, adolescent MA users show high rates of psychiatric symptoms [4, 9], especially depressive states [10], anti-social behaviour [11], and seem to retain psychiatric problems after prolonged abstinence [12].

In addition to comorbid psychiatric problems, a MUD in adolescence is associated with changes in brain structure and functioning. Specifically, adolescent MA users show reduced levels of n-acetylaspartic acid (NAA) in the prefrontal cortex (PFC), which indicates reduced neuronal integrity [13] and metabolic functioning of the PFC [14]. These brain changes might specifically influence cognitive performance, since lower levels of NAA in the frontal cortex have been shown to be related to reduced attention [15], executive functioning [16], and memory [17]. Additionally, the influence of MA use on the PFC could be particularly damaging in adolescence, since the PFC is still developing [18]. By interfering with this maturation process, MA use might disrupt the normal increase in inhibitory control or memory performance seen during adolescent development [19, 20].

Even though various lines of evidence point towards adolescent MA use being associated with cognitive impairments, few studies directly investigated cognitive functioning among adolescents with MUD [14, 21–23]. King et al. [22] found adolescents with a MUD show reduced performance in tasks related to inhibitory control, task-switching, spatial organization and fine motor speed [22]. Similarly, Cuzen et al. [21] found significant impairments in the domain of self-monitoring related to adolescent MA use [21]. On the other hand, Lyoo et al. [23] failed to detect significant differences in cognitive performance between MA and non-MA using adolescents. However, Kim et al. [14] used the same dataset and found MA-using adolescents to perform worse than non-using adolescents specifically in the Stroop task.

In conclusion, in adolescent MA users with MUD, only executive functions like inhibition and self-monitoring were consistently found to be impaired compared to non-MA using adolescents. However, these findings are in stark contrast to research with adults with MUD, who consistently show impairments in verbal learning, verbal memory, and short-term memory in addition to executive functions [6, 7, 24]. To further investigate what specific cognitive impairments are associated with MA use and MUD in adolescence we conducted the present study. Building upon previous studies [14, 21–23] we matched one sample of adolescents with a MUD, with one sample of adolescents with other SUDs, and one sample of adolescents without SUDs on age, gender, and depressivity. Those with MUD or SUD were additionally matched regarding their use of substances other than MA, i.e. cannabis, alcohol, and other stimulants (amphetamine or 3,4-methylendioxymethamphetamine (MDMA)). Additionally, we aimed to investigate how MA use frequency is associated with cognitive performance.

## Methods

### Participants

Between November 2017 and November 2020, *n* = 234 treatment-seeking adolescents at an outpatient clinic for adolescent substance abuse consented to participating in the study. Patients that i) fulfilled criteria for a MUD, and ii) had used MA in the past year on a regular basis (at least 1 day per month) were selected (“MA” group, *n* = 18). To control for co-occurring use of further substances, *n* = 18 participants were selected who presented at the clinic with other substance use disorders (SUDs) than MUD and had not used MA at all in the past year (“noMA”). See Additional Table 1 for an overview over SUDs in each group. Additionally, we recruited *n* = 18 control participants that did not report any past-year substance use or fulfilled criteria for any SUD (“noSUD”). In the final sample of *n* = 54 participants, the mean age was 16.1 years (*SD* = 1.2, range = 13.4–18.0 years) with 44% (*n* = 24) females. The three samples matched in terms of depressivity, age, and gender distribution while the MA and noMA groups also were matched on past year use of tobacco, alcohol, cannabis, MDMA (“ecstasy”), and amphetamine. The matching procedure was conducted across groups. While a 1-to-1 procedure would have been preferable, the participants reported highly individualized substance use patterns, preventing us from successful 1-to-1 matching. Matching resulted in comparable groups across all matching variables (all *p* > .199) as shown in Table 1.

**Table 1:** Group comparison between the analysis groups concerning depressivity, gender, age, and substance use frequency

	MA	noMA	noSUD	Group differences			Total
				*Test statistic (df)*	*p-value*	*Effect size*	
**N (female)**	18 (8)	18 (8)	18 (8)				54 (24)
**Mean age in years (*SD*)**	16.4 (1.2)	16.0 (1.1)	15.9 (1.3)	*F* [2, 51] = 0.968	.387	*d* = 0.41	16.1 (1.2)
**Mean BDI-II score (*SD*)**	19.1 (11.5)	16.3 (14.4)	11.0 (12.2)	*F* [2, 48] = 0.968	.199	*d* = 0.63	15.7 (12.9)
**Average monthly frequency of substance use (in days per month):**							
Tobacco (*SD, n*)	27.5 (7.7, *n* = 17)	25.0 (10.4, *n* = 16)	0.75 (2.8, *n* = 3)	*F* [1, 34] = 0.668	.419	*d* = 0.27	17.75 (14.3, *n* = 36)
Alcohol (*SD, n*)	4.7 (7.9, *n* = 14)	7 (10.1, *n* = 13)	0	*F* [1, 34] = 0.563	.458	*d* = 0.25	3.9 (7.8, *n* = 27)
Cannabis (*SD, n*)	19.9 (12.5, *n* = 18)	17.0 (12.2, *n* = 17)	0	*F* [1, 34] = 0.501	.484	*d* = 0.24	12.3 (13.3, *n* = 35)
Amphetamine (*SD, n*)	0.7 (1.4, *n* = 5)	0.3 (1.0, *n* = 3)	0	*F* [1, 34] = 0.839	.366	*d* = 0.31	0.3 (1.0, *n* = 8)
MDMA (*SD, n*)	3.4 (4.2, *n* = 14)	3.0 (7.6, *n* = 7)	0	*F* [1, 34] = 0.037	.848	*d* = 0.06	2.2 (5.2, *n* = 21)
MA (*SD, n*)	12.7 (13.0, *n* = 18)	0	0				4.2 (9.5, *n* = 18)

*Notes*: *d*, Cohen’s d with 0.2 being considered small, 0.5 medium, and 0.8 large [25]; *SD,* standard deviation; *BDI-II*, Beck Depression Inventory II; *MA*, methamphetamine; *MDMA*, 3,4-methylendioxymethamphetamine (“ecstasy”)

### Procedure

Data collection was imbedded into the recruitment and diagnostic procedure of a larger study conducted at our outpatient clinic (to be published, registered at clinicaltrials.govNCT03444974). Controls were recruited from age and gender matched patients without SUD as well as local advertisements for study participation. During the first appointment a trained clinical psychologist assessed the substance use variables in a structured interview and recorded age and gender of the participants. Informed consent of a legal guardian was obtained in this appointment as well. Cognitive testing took place in a subsequent appointment within 1 to 4 weeks.

To verify abstinence from stimulants (MDMA, amphetamine, MA), benzodiazepines, opiates, barbiturates, and cocaine in the past 24–72 h we performed urinary analysis (nal von minden Multi 10TT drug-screen) before the cognitive tests were conducted. If the urinary analysis was positive for any substance but tetrahydrocannabinol (THC) the cognitive testing was not performed. We allowed a positive THC urine screen due to the long time THC can be detected in urine compared to other substances, making it an unsuitable marker for acute substance-induced impairment. In the MA group, *n* = 2 participants screened positive for THC and *n* = 6 participants in the noMA group. The proportion of THC-positive drug screens did not differ between the two groups (*χ2* (1) = 2.57, *p* = .109, *OR* = 0.25 [0.04–1.46]). The study was conducted in accordance with the Declaration of Helsinki. All procedures were approved by the Institutional Review Board of the University Hospital C. G. Carus Dresden (EK 66022018).

### Measures

#### Depressivity

To assess and match participants on depressivity, we used the Beck Depression Inventory II (BDI-II) [26], a self-report questionnaire consisting of 21 questions. The BDI-II items cover the presence of various symptoms of depression in the past two weeks and are rated on a four-point scale ranging from 0 to 3, with a maximum total score of 63. Higher scores indicate higher depressivity.

#### Substance use

The extent of substance use was assessed by clinical psychologists or trained & supervised student assistants via a self-designed interview, asking the participants specifically for the average number of days each substance (alcohol, cannabis, MDMA, amphetamine, MA) was used per month over the past year. Age of first use per substance was recorded, allowing to categorize participants as lifetime abstainers per substance. Additionally, the interview included the assessment of SUD criteria for each substance according to DSM-5 [27].

#### Cognitive testing

Participants in this study performed six cognitive tests overall, providing data to analyse up to 30 different test outcomes. Having small sample sizes due to focussing on MUD which is comparably rare in adolescent SUD patients, it is recommendable to use a minimum number of outcome variables to reduce alpha error inflation [28]. The six tests mentioned above can be categorized as following: tests of inhibitory control (Stroop, Stop-Signal, Test of Attentional Performance (TAP) go/noGo), tests of attentional performance (TAP alertness, TAP divided attention) and tests of verbal memory (VLMT). We thus chose a-priori one test from each of the three assessed cognitive functions (verbal memory, inhibitory control, attention). This resulted in our selection of the VLMT (verbal memory performance), the TAP go/noGo (inhibitory control), and the TAP alertness (attention) tests. Following is a description of the selected tests and the outcomes we analysed from each test.

The manualized *Verbal Learning and Memory Test (VLMT)* [29] is a German version of the Rey Auditory Verbal Learning Task [30]. During the VLMT, participants listened to a list of 15 words being read out five times. During each turn, the participant had to remember and afterwards repeat as many words as possible (trials 1–5). Subsequently, a new list of 15 words was read out which participants also had to remember and repeat (interference). Directly after that participants had to recall as many words as possible from the first list (trial 6). After a 30-min break, in which the other cognitive tasks were performed, the participants were asked to repeat all words from the first list (trial 7). Finally, participants had to indicate if a word being read out loud was part of the first list or not (cued recall). Since this test includes over 10 possible outcomes, covering three distinct stages, we decided to choose only three outcomes, representing the different stages of the test and therefore different aspects of verbal memory: 1) number of words correctly recalled in *trial 1*, representing short-term memory, 2) number of words correctly recalled in *trial 5*, representing the ability to learn new information, 3) number of words correctly recognized from the list of words read-out loud, corrected by the number of false positives in this task (*cued recall)*, which can be considered a measure of longer term recall. In previous studies, the delayed recall variable (trial 7) is often used as a measure of longer term recall and consolidation [21]. However, we decided against using this outcome for two reasons. One, performance in trial 7 is highly dependent on performance on trial 5, meaning that a participant who had a low score in trial 5 would arguably also have a low score in trial 7. Therefore, a low score in trial 7 could either reflect low delayed recall ability or low ability to learn new information (trial 5) which makes an interpretation of trial 7 not straightforward. Second, to counter the first problem, sometimes a composite variable is created consisting of trial 7 being corrected for by performance in trial 5 (trail 5 minus trial 7). However, since we already included trial 5 as an outcome on its own this would lead to an interdependence of our outcome variables which we wanted to avoid. Therefore, we decided to use cued recall as a measure of longer term verbal memory.

The *Test of Attentional Performance (TAP)* is a comprehensive test battery assessing various cognitive domains related mainly to attentional performance [31]. Three subtests of this battery were administered and two (“alertness”, “go/noGo”) were analysed here as measures of intrinsic alertness and inhibitory control. Intrinsic alertness refers to the ability to maintain an optimal level of arousal for a short time and is the building block for more complex cognitive functions [32]***.*** To assess intrinsic alertness, we recorded the mean reaction time in milliseconds of participants pressing a button in reaction to a visual stimulus (subtest “alertness”). The “go/noGo” task assesses *inhibition* instead of other executive functions like *updating* or *shifting* [33]. The task consists of two similar-looking different visual stimuli, in reaction to one of which participants have to press a button, and for the other withhold the response. As an outcome for this task we computed an inverse efficiency score (IES) [34] by dividing the mean reaction time for correct go trials divided by the proportion of correct response to no-go trials. A higher IES represents lower inhibition competencies.

Overall, this test selection results in five cognitive variables on which to compare the groups: trial 1, trial 5, cued recall, alertness, go/noGo. Since the VLMT involves a 30-min break between trial 6 and trial 7, participants were presented with the TAP subtests in this timeframe.

### Statistical analysis

Since the majority of our five outcome variables (trial 1, trial 5, cued recall, alertness, go/noGo) were not normally distributed in at least one group (see Additional Table 2), we performed a logarithmic transformation of the non-normally distributed outcomes (trial 1, trial 5, cued recall). However, since the log-transformed outcomes were also distributed not normally (see Additional Table 3), we performed a non-parametric Kruskal-Wallis test to check for differences across all three groups (MA, noMA, noSUD). In case the omnibus test was significant we performed post-hoc Mann-Whitney U tests to check for specific differences between all three groups. Additionally, we calculated Spearman’s rank order correlations between the average number of days of MA use per month in the past 12 months and cognitive test scores. To correct for type 1 errors through multiple testing we used a Bonferroni correction [35] in the following ways: For the omnibus test we divided the significance level of .05 by the number of outcome variables (= 5), resulting in a threshold *p*-value of .01. For the post hoc analysis we divided the significance level of .05 by the number of post hoc comparisons (= 3), resulting in a threshold p-value of .016. For the Spearman’s rank order correlational analysis, we divided the significance level of .05 by the number of correlations of interests to us (all correlations of MA with the outcome variables = 5), resulting in a threshold p-value of .01. Effect sizes were classified according to Cohen [25] into small effects (|r | ≥ .10, | η_p_^2^| ≥ .01), medium effects (|r | ≥ .30, | η_p_^2^| ≥ .06), and large effects (|r | ≥ .50, | η_p_^2^| ≥ .14). Partial eta squared (η_p_^2^) is calculated by dividing the effect specific sum of squares by the sum of the effect sum of squares and error related sum of squares (η_p_^2^ = SSeffect / SSeffect + SSerror). All analyses were conducted with IBM SPSS Statistics for Windows, version 27.0 [36].

## Results

### Overall differences

The three groups (MA, noMA, noSUD) differed significantly in their performance on trial 5, and this was the only difference reaching a large effect size (*H* [2] = 11.7; *p* = .003; *η*_*p*_^*2*^ = .19). All other variables (trial 1, cued recall, go/noGo, alertness) did not differ significantly between groups (all *p* >.01; all *η*_*p*_^*2*^ < .06). Mean scores and results of the omnibus test for all cognitive domains are displayed in Table 2.

**Table 2:** Mean scores and group comparison on five cognitive variables

	MA (***n*** = 18)	noMA (***n*** = 18)	noSUD (***n*** = 18)	Group differences		
				*Test statistic (df)*	*p-value*	*Effect size*
**Trial 1**	6.1 (2.5)	6.8 (2.0)	7.7 (2.1)	*H* [2] = 2.7	.259	*η*_*p*_^*2*^ = .014
**Trial 5**	10.8 (2.8)	12.5 (2.2)	13.6 (1.6)	*H* [2] = 11.7	.003**	*η*_*p*_^*2*^ = .190^++^
**Cued recall**	12.0 (3.3)	12.6 (2.9)	13.5 (1.5)	*H* [2] = 0.9	.623	*η*_*p*_^*2*^ = .022
**Go/noGo**	468.1 (65.6)	452.4 (56.2)	451.8 (60.2)	*H* [2] = 3.3	.188	*η*_*p*_^*2*^ = .025
**Alertness**	273.0 (50.7)	249.3 (34.8)	256.5 (21.1)	*H* [2] = 1.4	.493	*η*_*p*_^*2*^ = .011

*Notes:* ** *p* < 0.01; ^++^ large effect as indicated by η_p_^2^ > 0.13 [25]. *Trial 1,* number of words recalled on trial 1 of the VLMT; *trial 5,* number of words recalled on trial 5 of the VLMT; *cued recall,* number of words of the VLMT correctly recognized after a delay, corrected for recognition mistakes; *go/noGo,* inverse efficiency score of the “go/noGo” subtest of the TAP; *alertness,* mean reaction time of the “alertness” subtest of the TAP in milliseconds; *VLMT*, Verbal Learning Memory Test; *TAP*, Test of Attentional Performance

### Post hoc analysis

**Fig. 1:**
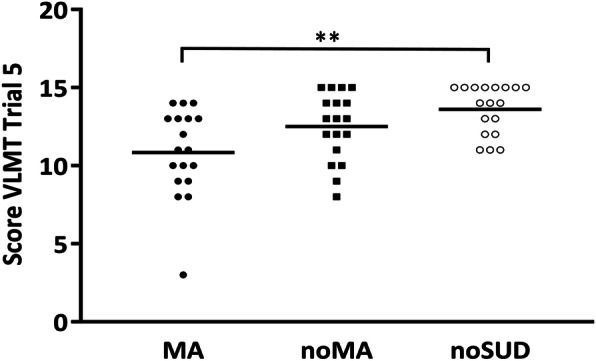
Distribution of verbal learning scores, with one symbol equal to one participant. The black line represents the mean score in each group. ** *p* < 0.016; *VLMT,* Verbal Learning and Memory task; *MA;* methamphetamine using groups; *noMA;* group using substances but not methamphetamine; *noSUD;* group that uses no psychoactive substances

Post hoc Mann-Whitney U tests for trial 5 found the following results: The MA group scored significantly lower than the noSUD group (*U* = 56.5, *p* = .001, *η*_*p*_^*2*^ = .31) with a large difference. The MA group scored also lower than the noMA group with a medium-sized effect. However, the difference was not significant (*U* = 104, *p* = .068, *η*_*p*_^*2*^ = .09). Similarly, the noMA group scored lower than the noSUD group with a medium-sized effect that did not reach significance. (*U* = 113, *p* = .126, *η*_*p*_^*2*^ = .07). Score distributions are displayed in Fig. 1.

### Associations of cognitive performance and substance use

Across all participants (*n* = 54), the number of days of MA use per month showed a significant and negative correlation of medium size with performance in trial 1 (*ρ* = −.25, *p* < .001) and trial 5 (*ρ* = −.41, *p* = .004) of the VLMT. No other cognitive variable correlated significantly with MA use frequency. All correlation coefficients are displayed in Table 3.

**Table 3:** Bivariate Spearman rank-order correlation values (ρ) between substance use frequency in the past year and cognitive variables

	Alcohol	Cannabis	Amphetamine	MDMA	MA
**Trial 1**	−.25	−.15	−.11	.06	−.25
**Trial 5**	−.39	−.39	−.02	−.33	−.41**
**Cued recall**	−.27	−.29	.08	−.23	−.14
**Go/noGo**	.08	.16	.01	..10	.13
**Alertness**	.02	.03	−.07	.21	.23

Notes: ** p < 0.01; trial 1, number of words recalled on trial 1 of the VLMT; *trial 5,* number of words recalled on trial 5 of the VLMT; *cued recall*, number of words of the VLMT correctly recognized after a delay, corrected for recognition mistakes; *go/noGo,* inverse efficiency score of the “go/noGo” subtest of the TAP; *alertness*, mean reaction time of the “alertness” subtest of the TAP in milliseconds; *VLMT*, Verbal Learning Memory Test; *TAP*, Test of Attentional Performance; *MDMA*, 3,4-methylenedioxymethamphetamine (“ecstasy”); *MA*, methamphetamine

## Discussion

In this cross-sectional study, we compared a group of adolescents with MUD with a group without MUD matched for depressivity, age, gender, and other substance use. Additionally, we compared both groups with adolescents without past-year substance use or SUDs, matched for depressivity, age and gender. We could show that adolescents with a MUD showed a reduced performance in trial 5 of the VLMT, indicating lower verbal learning ability. Additionally, a larger number of MA use days per month was associated with reduced performance in VLMT trials 1 and 5, indicating a negative association with short-term memory and verbal learning ability respectively. The negative relationship between performance in trial 5 and MA use was confirmed by the significant negative correlation, as well as medium to large differences between the MA and noMA group as well as the MA group and noSUD group.

Our results are in line with research in adult MUD patients in which medium sized negative effects are found for verbal learning and verbal memory [7]**.** Specifically, previous studies in adults also associated MA use with learning impairments in an auditory verbal learning task (AVLT) [37–40]. Further, Hoffman et al. [37] found a significant negative association between MA use and performance on the first trial of an AVLT as well, which supports the finding of our correlational analysis. Two biological processes related to MA use might explain the effects of MA use on verbal memory performance. First, regular use of MA seems to diminish functionality of n-methyl-d-aspartate (NMDA) and quisqualate (AMPA) receptors in the striatum and frontal cortex through an MA-induced increase of glutamate flow [41]. These effects [41, 42], in addition to the MA-induced dysfunction of NAA in the PFC [13, 14], might play a role in the observed verbal learning deficits, since normal PFC development is related to increases in memory performance [17, 18]. Additionally, high doses of MA have been shown to lead to neuronal damage in the mouse hippocampus [43], which is a region strongly involved in memory processing [44].

Second, MA use is associated with reduced density of the dopamine transporter [45], which is directly related to memory impairments, specifically in an AVLT [39]. Volkow et al. [39] used PET scanning to show that the presence of a MUD is related to decreased dopamine transporter availability in the striatum, which was directly related to verbal learning impairment. Whereas the loss of dopamine transporters seems to recover with MA abstinence, verbal learning ability has shown no such recovery in adults [46].

While consistent with findings in adult MA users, our results oppose previous studies in adolescents [21–23]. All three studies also used an AVLT to assess memory performance but detected no significant differences between MA using and non-using groups [21–23]. However, we detected a medium-sized negative effect for verbal learning performance and significant negative associations between MA use and short-term memory as well as verbal learning. A possible explanation for this difference in results might be the selection of outcome variables. We used three specific variables from the VLMT on which to compare participants and in doing so focused on specific aspects of verbal memory (short-term memory, learning ability, cued recall). In contrast, Lyoo et al. [23], and Cuzen et al. [21], calculated new variables across several tests to compare groups on global domain scores. If they had included comparisons between singular variables, focusing on specific aspects of verbal memory instead of the global domain, similar differences as we found might have emerged. Additionally, King et al. [22] sampled adolescent with a MUD that were abstinent for several months at the time of testing. While abstinence-related recovery of memory performance has not been shown for adults [46, 47], no investigations in adolescent users have been conducted. It is possible, that the abstinent adolescent MA users had already recovered memory performance at the time of testing. However, we cannot be sure if the difference in memory outcomes is due to a possible recovery effect or that King et al. [22] did not control for use of other stimulant-type drugs (e.g., amphetamine or MDMA). Especially the second aspect is important, since use of other stimulant-type drugs has also been shown to be related to impairments in verbal memory [48].

Another discrepancy to previous research relates to inhibitory performance. Four previous investigations with adolescents [14, 21–23] found a negative effect of MA use on inhibitory control and self-monitoring, while we did not. One explanation for the discrepancy could be test selection. All studies with adolescents [14, 21–23] as well as the majority of adult studies [24] assessed inhibition with the Stroop task, while we used a go/noGo task. Even though both tests load on an factor related to inhibition [49], the Stroop task is mainly a measure of taking control over an interference effect, while the go/noGo task measures the inhibition of an activated motor response [50]. Thus, the combination of previous research in adolescents and our results indicates that MA use in adolescents might be uniquely related to impairments in interference control, rather than pure response inhibition.

### Limitations

First, we recruited a small sample, which constrains the generalizability of our results. Nonetheless, sample sizes of this magnitude are common in studies dealing with MUD patients, e.g. 9 of the 17 studies investigating cognitive functioning in MA users included in the review by Scott et al. [24] had groups of MA users with *n* < 20. Furthermore, we took great care to control for various confounding factors by applying an extensive matching procedure.

Second, our sample consisted of adolescents with MUD that consumed other psychoactive substances on a regular basis. On one hand, this is an accurate representation of the reality of adolescent MA users and we took great effort to match the groups on their substance use. On the other hand, combining MA use with other substances might have additive detrimental effects on cognitive performance over and above MA use on its own. For example, Cuzen et al. [21] showed that users of MA and cannabis showed stronger cognitive deficits, than exclusive MA users. Third, we did not record the time of abstinence since last MA use. Since, it might be possible that cognitive effects recuperate after MA abstinence (see [22, 51]), future longitudinal studies need to control for this variable.

## Conclusions

This is the first cross-sectional study assessing cognitive impairments in adolescent MA users with a MUD, while specifically controlling for the use of other stimulant-type drugs. We could show that the presence of a MUD is specifically related to a verbal learning impairment. Additionally, frequency of MA use was negatively associated with verbal learning and short-term memory. Further research should aim to recruit adolescents who use only MA and assess cognitive domains with more detailed test batteries, while also controlling for time of MA abstinence. Clinicians working with adolescents with MUD should take care to adapt their interventions to the cognitive abilities of their patients.

## Supplementary Information

**Additional file 1 Table 1.** Number of participants in the MA and noMA group fulfilling the criteria for various substance use disorders. **Table 2.** Shapiro-Wilk test for normality of the five cognitive outcome variables. **Table 3.** Shapiro-Wilk test for normality of the three log-transformed cognitive outcome variables.

## Data Availability

The datasets used and analysed during the current study are available from the corresponding author on reasonable request.

## Abbreviations

MA: Methamphetamine
MUD: Stimulant use disorder – methamphetamine type
SUD: Substance use disorder
MDMA: 3,4-methylendioxymethamphetamine
THC: tetrahydrocannabinol
PFC: Prefrontal cortex
NAA: N-acetylaspartic acid
AVLT: Auditory verbal learning task
VLMT: Verbal Learning and Memory Task
TAP: Test of Attentional Performance
IES: Inverse efficiency score
