# Supplementary material for: Verbal learning impairment in adolescents with methamphetamine use disorder: a cross-sectional study
Source: BMC Psychiatry. 2021 Mar 25;21:166. doi: 10.1186/s12888-021-03169-3 (PMC7993453; doi:10.1186/s12888-021-03169-3)
Supplement: Supplementary file 1 — Additional file 1 Table 1. Number of participants in the MA and noMA group fulfilling the criteria for various substance use disorders. Table 2. Shapiro-Wilk test for normality of the five cognitive outcome variables. Table 3. Shapiro-Wilk test for normality of the three log-transformed cognitive outcome variables. [file 12888_2021_3169_MOESM1_ESM.docx]

*Verbal learning impairment in adolescents with methamphetamine use disorder: a cross-sectional study*

*Authors:* Lukas Andreas Basedow^1^, Sören Kuitunen-Paul^1^, Melina Felicitas Wiedmann^1^, Stefan Ehrlich^1,2^, Veit Roessner^1^, Yulia Golub^1^

Additional Files

*Additional Table 1.* Number of participants in the MA and noMA group fulfilling the criteria for various substance use disorders.

|  | **MA**  (n = 5 missings | **noMA**  (n = 1 missing) | **Total**  (n = 6 missing) |
| --- | --- | --- | --- |
| **Alcohol use disorder** | 4 | 9 | 13 |
| **Cannabis use disorder** | 11 | 12 | 24 |
| **Stimulant use disorder – MDMA type** | 7 | 4 | 11 |
| **Stimulant use disorder – amphetamine type** | 2 | 0 | 2 |
| **Multiple substance use disorders** | 12 | 8 | 20 |

*Notes*: *MDMA*, 3,4-methylendioxymethamphetamine (“ecstasy”).

*Additional Table 2.* Shapiro-Wilk test for normality of the five cognitive outcome variables.

|  | **MA** | | **noMA** | | **noSUD** | |
| --- | --- | --- | --- | --- | --- | --- |
|  | *Test statistic* | *p-value* | *Test Statistic* | *p-value* | *Test Statistic* | *p-value* |
| **Trial 1** | W(18) = .887 | .035* | W(18) = .930 | .193 | W(18) = .812 | .002* |
| **Trial 5** | W(18) = .888 | .036* | W(18) = .917 | .116 | W(18) = .800 | .002* |
| **Cued recall** | W(18) = .819 | .003* | W(18) = .785 | .001* | W(18) = .865 | .014* |
| **go/noGo** | W(18) = .985 | .986 | W(18) = .925 | .157 | W(18) = .958 | .555 |
| **Alertness** | W(18) = .891 | .040* | W(18) = .914 | .102 | W(18) = .971 | .807 |

*Notes:* *p < 0.05; *trial 1,* number of words recalled on trial 1 of the VLMT; *trial 5*, number of words recalled on trial 5 of the VLMT; *cued recall*, number of words of the VLMT correctly recognized after a delay, corrected for recognition mistakes; *go/noGo,* inverse efficiency score of the “go/noGo” subtest of the TAP; *alertness*, mean reaction time of the “alertness” subtest of the TAP in milliseconds; *VLMT*, Verbal Learning Memory Test; *TAP*, Test of Attentional Performance

*Additional Table 3.* Shapiro-Wilk test for normality of the three log-transformed cognitive outcome variables.

|  | **MA** | | **noMA** | | **noSUD** | |
| --- | --- | --- | --- | --- | --- | --- |
|  | *Test statistic* | *p-value* | *Test Statistic* | *p-value* | *Test Statistic* | *p-value* |
| **Trial 1** | W(18) = .717 | >.001* | W(18) = .946 | .372 | W(18) = .887 | .034* |
| **Trial 5** | W(18) = .755 | >.001* | W(18) = .897 | .051 | W(18) = .794 | .001* |
| **Cued recall** | W(18) = .768 | .001* | W(18) = .650 | >.001* | W(18) = .850 | .008* |

*Notes:* *p < 0.05; *trial 1,* number of words recalled on trial 1 of the VLMT; *trial 5*, number of words recalled on trial 5 of the VLMT; *cued recall*, number of words of the VLMT correctly recognized after a delay, corrected for recognition mistakes; *VLMT*, Verbal Learning Memory Test
